# Recent insights on functional heartburn and reflux hypersensitivity

**DOI:** 10.1097/MOG.0000000000000846

**Published:** 2022-06-24

**Authors:** Edoardo Savarino, Elisa Marabotto, Vincenzo Savarino

**Affiliations:** a Gastroenterology Unit, Department of Surgery, Oncology and Gastroenterology, University of Padua, Padua; b Gastroenterology Unit, Department of Internal Medicine and Medical Specialties, University of Genoa, Genoa, Italy

**Keywords:** baseline impedance, functional heartburn, gastroesophageal reflux disease, postreflux swallowing peristaltic wave, reflux hypersensitivity

## Abstract

**Purpose of review:**

Rome IV experts have proposed that gastroesophageal reflux disease (GERD) should be diagnosed only in patients with abnormal esophageal acid exposure, and that reflux hypersensitivity (RH) and functional heartburn (FH) both should be considered functional conditions separate from GERD. Although past and recent evidence support that FH can be completely distinguished from GERD, the concept that RH is not GERD is highly questionable. This review attempts to provide current data on these issues.

**Recent findings:**

Many recent investigations have provided new data on the different pathophysiological features characterizing RH and FH. Major differences have emerged from analyses of impedance-pH monitoring studies using the novel impedance metrics of baseline impedance (an index of mucosal integrity) and the rate of postreflux swallow-induced peristaltic waves (a reflection of the integrity of esophageal chemical clearance).

**Summary:**

The better ability to interpret impedance-pH tracings together with earlier data on the different prevalence of microscopic esophagitis in RH and FH patients, and recent studies documenting poor therapeutic efficacy of pain modulators and good results of antireflux surgery for RH support recategorization of RH within the GERD world. Further research is needed to correctly phenotype patients who have heartburn without mucosal breaks, and to guide their effective management.

## INTRODUCTION

For many years gastroesophageal reflux disease (GERD) was equated with erosive esophagitis (EE), and the majority of clinical trials aimed at assessing the efficacy of proton pump inhibitors (PPIs), the most potent antisecretory drugs) were performed in patients with reflux esophagitis. However, in the two past decades, we have realized that the majority (∼70–80%) of patients with heartburn do not have esophageal mucosal lesions detectable at endoscopy [1] and, for these cases, the term nonerosive reflux disease (NERD) was created [2].

Subsequently, various pathophysiological studies using the most valid diagnostic tool represented by 24-h impedance-pH monitoring have enabled us to understand that the NERD population is markedly heterogeneous and can be subdivided into several well-defined subgroups with specific functional characteristics [3]: (a) patients with true NERD with an excess of acid burden within the esophagus; (b) patients with mucosal hypersensitivity to acid or weakly acidic reflux despite having physiologic levels of acid reflux and; (c) patients with functional heartburn (FH) who have normal esophageal acid exposure with no association between reflux episodes and reflux symptoms.

The Rome III criteria, developed for the definition of functional esophageal disorders [4], included in their definition of GERD both NERD patients with increased esophageal acid exposure time (AET) and those with hypersensitive esophagus (HE) on the basis of the evidence of symptom-reflux association despite normal AET, using a pH study and/or the symptom relief from a PPI trial. However, the most recent Rome IV criteria [5] proposed that HE should be renamed reflux hypersensitivity (RH) and should not be included within the GERD population, but rather should be classified in the realm of functional esophageal disorders along with, but separate from FH.

In other words, the Rome IV experts concluded that factors other than acid can be responsible for the generation of heartburn, and that visceral hypersensitivity seems to be the predominant mechanism underlying heartburn in patients with RH, who have physiologic AET. Furthermore, they concluded that response to PPI therapy is an unreliable criterion for RH. If this is the case, then the modulating effects of hypervigilance, visceral and central hypersensitivity need to be recognized in order to guide our therapeutic approach to patients who have typical reflux symptoms without endoscopic mucosal damage [6^▪▪^]. The physiologic determinants of enhanced esophageal sensitivity include abnormalities of mucosal integrity, notably dilated intercellular spaces (DIS) [7], and lower stimulation thresholds for the TRPV1 receptors or acid sensing ion channels in the esophageal mucosa [8] as well as alterations in central pain perception, which can be assessed by functional brain magnetic resonance imaging [9]. The proximal extent of reflux and the presence of gas mixed with liquid in the refluxate have also been implicated in perception of symptoms in NERD [10,11]. Lastly, patient anxiety levels and psychological co-morbidities seem to play additional relevant roles in potentiating the perception of intra-esophageal stimuli [12]. Indeed, recent studies have recommended the use of innovative questionnaires, like the esophageal hypervigilance and anxiety scale (EHAS, which is a validated cognitive-affective evaluation of centrally mediated esophageal symptom perception) to measure the contribution of anxiety and other affective disorders to the perception of esophageal symptoms [13,14^▪^].

Therefore, the practice of treating all patients presenting with the symptom of heartburn with PPIs, as is done typically in both western and eastern countries, should be avoided. Instead, the use of drugs directed at controlling the specific pathophysiological features of each GERD syndrome should be adopted [15^▪▪^].

Rome IV criteria also have proposed a further functional subgroup in addition to RH and FH, as it has been hypothesized that these two functional subgroups could overlap with true GERD, identified by the findings of positive reflux monitoring, EE, Barrett esophagus or peptic strictures in patients with persisting heartburn despite PPI therapy, provided that acid burden is controlled by PPIs [5]. A recent prospective study has shown that conventional and GERD-overlap FH and RH have similar clinical, psychological, and functional profiles, thus giving support to the Rome IV hypothesis [16^▪^]. However, some limitations of this study have been acknowledged by the authors, such as the low generalizability of the conclusions, the small sample size, and the lack of management impact on symptoms and outcomes, suggesting that further investigations are necessary. A previous study [17] on patients refractory to once-daily PPIs did not find any significant differences in impedance-pH parameters between GERD patients who responded and those who failed to respond to PPI therapy and concluded that there can be considerable overlap of well documented GERD with functional esophageal disorders, specifically 62.5% with FH and 12% with RH. However, this study also had important limitations, mainly its small sample size and the use of a single daily dose of PPIs. 

**Box 1:**
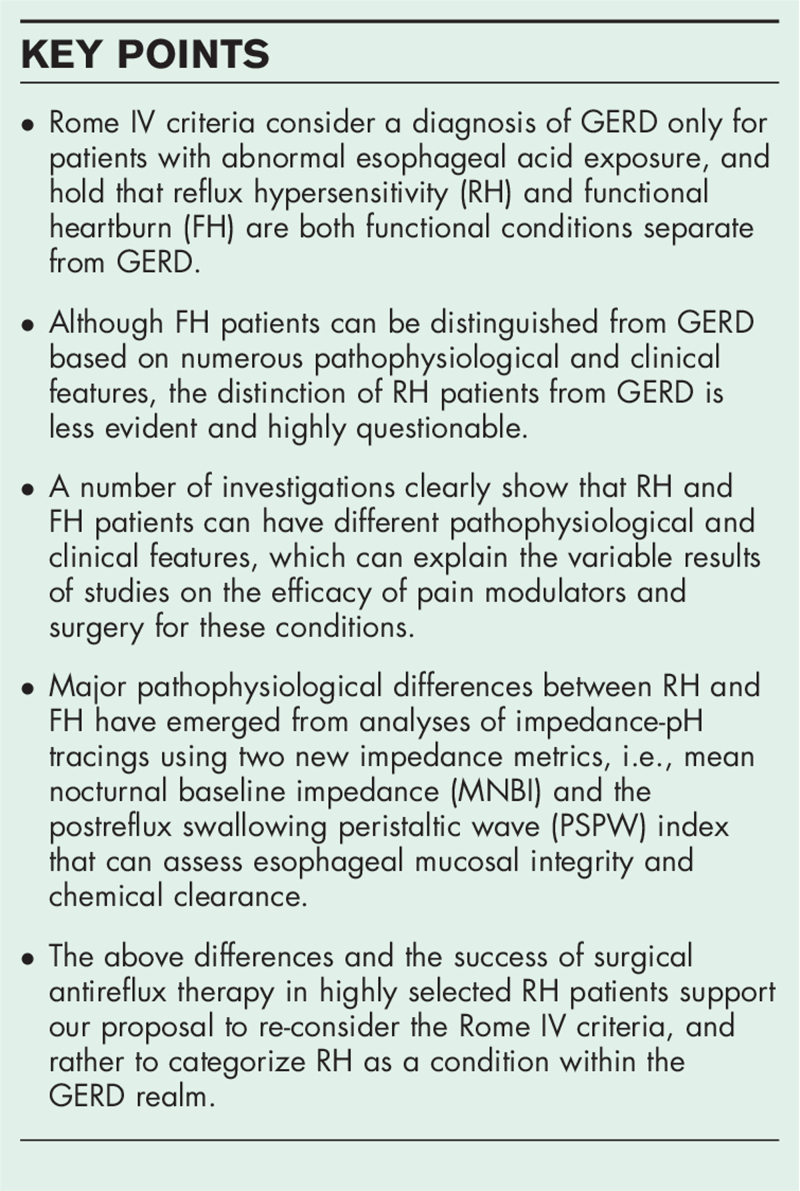
no caption available

## FUNCTIONAL HEARTBURN DIFFERS FROM NONEROSIVE REFLUX DISEASE/GASTROESOPHAGEAL REFLUX DISEASE

Various pathophysiological and clinical studies have shown that FH is not the same as NERD, although these patients share the same symptom heartburn with no differences in terms of frequency and intensity. In fact, patients with NERD have a higher prevalence of hiatal hernia, lower baseline tone in the lower esophageal sphincter (LES), higher acid burden, and more abnormalities in mucosal integrity and chemical clearance (as determined by new impedance metrics such as the mean nocturnal baseline impedance (MNBI) and the postreflux swallow-induced peristaltic wave (PSPW) index) than patients with FH [18,19]. Moreover, studies with esophageal balloon distension and acid perfusion have documented lower pain perception thresholds in FH than in NERD patients [20]. Lastly, concomitant functional disorders, like functional dyspepsia (FD) and irritable bowel syndrome (IBS), have been found to be significantly more often in FH than in NERD [21,22].

FH patients do not respond to PPI therapy and, therefore, they should be treated with medications that can reduce visceral hypersensitivity and improve co-existing psychological dysfunction [23^▪^].

Considering the above pathophysiological and clinical differences between FH and GERD patients, it is evident that FH patients must be excluded from the GERD world since gastro-esophageal reflux does not trigger the heartburn of FH. Instead, visceral hypersensitivity plays the main pathogenetic role [24] and studies using balloon distension in both the esophagus and the rectum of FH patients have shown a similar degree of visceral hypersensitivity, thereby confirming a generalized increase in perception of visceral stimuli [25]. More importantly, the rate of finding histologic changes of reflux esophagitis, in particular DIS, in FH patients is similar to that of healthy volunteers (HV) and much lower than that of NERD and EE patients [26], thus confirming the integrity of the esophageal mucosa in FH patients. There is also a higher likelihood of anxiety and other affective disorders in FH than in NERD patients [27^▪▪^].

All the above-mentioned features clearly indicate that FH is a separate entity from NERD and therefore GERD, and should be considered a distinct, functional disorder.

## REFLUX HYPERSENSITIVITY DISTINCTION FROM GASTROESOPHAGEAL REFLUX DISEASE: A REAL CHALLENGE

If the distinction between FH and GERD is clear-cut, the complete separation of RH from NERD is less evident and highly questionable. It is noteworthy that the Rome IV experts suggest changing the categorization of RH from NERD to a functional esophageal disorder, but continue to maintain a distinction between RH and FH. Indeed, there are many reasons to separate RH from FH, as shown in Table 1, but there is some important recent evidence that would support re-consideration of RH as a GERD subgroup [28^▪▪^].

**Table 1:** Main histologic, pathophysiological and clinical differences between RH and FH patients studies off-PPI therapy.

	Reflux hypersensitivity	Functional heartburn
Microscopic esophagitis [26]	65%	13%
Weakly acidic reflux [26,32]	Increased	Normal
Proximal reflux migration [26,32]	Increased	Normal
Mean nocturnal baseline impedance (MNBI) [35–37,39]	Low values	Normal values
Postreflux swallow-induced peristaltic wave (PSPW) index [35–37]	Low values	Normal values
Supra-gastric belching or rumination[40^▪^]	Possible overlap	No overlap
Anxiety level [27^▪▪^,^41^]	Similar to other GERD subsets	High rate
Response to PPIs [28^▪▪^]	Possible benefit	No benefit
Response to antireflux surgery [46–50]	Possible benefit	No benefit

FH, functional heartburn; GERD, gastroesophageal reflux disease; MNBI, mean nocturnal baseline impedance; PPIs, proton pump inhibitors; RH, reflux hypersensitivity.

First, as noted above, microscopic esophagitis, including DIS, is a common finding in patients who have reflux symptoms with negative endoscopies, with a different distribution of this histopathological entity across the various subsets of this population [26]. In fact, we have found that microscopic esophagitis occurs with considerably less frequency in controls (15%) and FH patients (13%) than in RH patients (65%) or in those with increased AET (77%). This significantly greater microscopic damage in RH and pH-positive NERD than in FH may be responsible for symptom generation in the former two subgroups because it can enable the components of refluxate to reach and activate esophageal chemo-sensitive receptors.

In addition, many pathophysiological studies performed with modern impedance-pH monitoring have shown important differences between RH and FH. For instance, Savarino *et al.*[29] confirmed a higher AET in RH than in FH, and reported a significantly increased number of weakly acidic reflux events and a higher rate of proximal reflux than in FH and HV. It has been hypothesized that the increased number of weakly acidic reflux episodes may induce microscopic damage to the esophageal mucosa [26], as mentioned above, resulting in sensitization of the esophagus and heightened perception of reflux events, as supported by the high rate of symptoms associated with weakly acidic reflux events in RH patients [30]. The higher percentage of reflux episodes reaching the proximal esophagus may also contribute to elicit reflux symptoms, as reported in other studies [11,31]. More recently, Gao *et al.*[32] confirmed that Chinese patients with RH have greater acid exposure and total bolus exposure time as well as more proximal and distal reflux events than FH patients, despite the presence of physiologic levels of acid burden in both subgroups.

The difference between RH and FH is even more evident when using the new impedance metrics of MNBI and PSPW index, both of which have been shown to improve the diagnostic yield of impedance-pH monitoring for GERD [19]. Low MNBI is an expression of impaired esophageal mucosal integrity, whereas a low PSPW index reflects the impaired esophageal peristalsis and chemical clearance that often are associated with GERD [33^▪^]. A recent study [34^▪▪^] comparing conventional and new impedance-pH parameters showed that the latter substantially increase the diagnostic yield of reflux monitoring because they can confirm a GERD diagnosis in approximately one-third of patients who have normal AET values.

Some studies have found that MNBI and PSPW index are significantly lower in RH than in FH patients [35,36], thus confirming the presence of reduced mucosal integrity and poor reflux clearance in the former population. Sun *et al.*[37] confirmed that both MNBI and PSPW were significantly lower in RH than in FH Chinese patients. Furthermore, MNBI shows that FH patients responding to PPIs have lower baseline impedance values than those refractory to PPIs and similar to RH patients [38]. Finally, the combined use of MNBI and PSPW index increases the separation between RH and FH, affording an excellent area under the curve (> 0.90) on Receiver operating characteristic analysis and thus confirming that FH and RH are different populations [36]. The impairment of mucosal integrity, which is similar in RH and GERD patients and absent in FH patients [35,36,39] has been proposed to explain the positive response of RH patients to both medical (PPI) and surgical treatment [28^▪▪^].

It must be emphasized that RH patients are not a homogeneous population. A subset of RH patients seem to have reflux symptoms generated by behavior disorders, such as supra-gastric belching (SPG) or rumination [40^▪^], and should be treated with non-GERD therapeutic approaches, like diaphragmatic breathing or cognitive-behavioral therapy. These non-GERD, behavioral subgroups might explain why many RH patients fail to respond not only to PPIs, but also to pain modulators. Thus, the segregation of these non-GERD RH patients from those with FH might have important therapeutic implications. On the other hand, the lack of a perfect tool to diagnose with certainty the various phenotypes of patients with typical GERD symptoms makes it difficult to categorize patients reliably into subsets of functional esophageal disorders. In addition, this fact also calls into question the Rome IV assumption that RH is caused exclusively by visceral hypersensitivity. Only the future availability of more accurate diagnostic techniques will enable a more precise understanding of the complexity of GERD syndromes and better refinements in the diagnosis of both RH and FH.

Finally, as additional confirmation of the close relationship between RH and GERD, Kessing *et al.*[41], using ambulatory impedance-pH monitoring and a validated scale to assess anxiety and depression levels, found that RH patients had similar levels of anxiety, depression, and quality of life as patients with GERD, whereas FH patients had higher anxiety than all the other subgroups.

The Rome IV proposal to relegate two different conditions (RH and FH) into the category of functional disorders was based on the assumption that visceral hypersensitivity is the common pathophysiological mechanism underlying both conditions. However, clinical trials using neuromodulators to reduce visceral hypersensitivity in patients who had heartburn without EE have yielded conflicting results, with some [42,43] showing benefits of such therapy, and others [44,45] failing to show symptom improvement with results comparable to placebo.

In contrast, attempts to control both acid and weakly acidic reflux with surgical therapy have provided good results both in the short and in the long term in the RH population [46–49]. Although many of these studies were uncontrolled, Spechler *et al.*[50] have recently confirmed the benefit of surgery in a prospective, randomized and controlled trial, in which preoperative impedance-pH monitoring was used to detect the different GERD phenotypes, and laparoscopic fundoplication at 1 year resulted in significantly better control of reflux symptoms (67%) than active medical treatment (28%) and control medical treatment (12%) in patients for whom impedance-pH monitoring confirmed a positive reflux-symptom association. Therefore, this study provided important confirmation of the success of antireflux surgery in patients with RH, supporting the need for accurately identifying these RH patients and distinguishing them from FH patients by impedance-pH testing before surgery. Also, two international consensus groups recommended laparoscopic fundoplication as the treatment of choice in PPI-refractory GERD when the preoperative work-up clearly identifies RH [51,52]. On the other hand, it is well known that the benefit of surgery is poor in FH [53,54] and surgical therapy must be avoided as a therapeutic option for these patients. Finally, the recent 2020 Seoul consensus on the diagnosis and management of GERD concluded that RH should be considered a form of GERD [55^▪^].

Overall, the above-mentioned surgical success supports the concept that reflux events, both acid and weakly acidic, play a significant role in eliciting symptoms in RH patients and, therefore, corroborate the need of maintaining them within the GERD realm.

## CONCLUSION

In the last two decades, we have realized that GERD is an umbrella term encompassing various subpopulations with different pathophysiological features that must be taken into account if one is to adopt the correct therapeutic approach. The scant benefit of neuromodulators in the limited number of clinical trials performed in patients with functional esophageal disorders might reflect our poor understanding of the mechanisms underlying reflux symptom generation, and the complexity of the various subgroups of NERD, which can include patients with reflux symptoms due to non-GERD disorders, such as SGB or rumination. Future studies using more sophisticated techniques will be of help in segregating more accurately the various and complex subsets of patients who have heartburn without mucosal erosions.

It is now evident that the term ’NERD’ comprises many different syndromes that have distinct pathophysiological features requiring tailored approaches to management. The one-size-fits-all, traditional definition of endoscopy-negative reflux disease should be abandoned in favor of more specific diagnostic terms to avoid confusion and erroneous therapeutic decisions.

## Acknowledgements

*Authors’ contribution: E.S., E.M., V.S.: design of the study, data collection, writing of the manuscript, approving final version; E.S., E.M., V.S.: data collection, writing of the manuscript, approving final version.*

*Guarantor of the article: V.S.*

### Financial support and sponsorship

*None.*

### Conflicts of interest

*Potential competing interests: E.S. has served as speaker for Abbvie, AGPharma, Alfasigma, EG Stada Group, Fresenius Kabi, Grifols, Janssen, Innovamedica, Malesci, Pfizer, Reckitt Benckiser, Sandoz, SILA, Sofar, Takeda, Unifarco; has served as consultant for Alfasigma, Amgen, Biogen, Bristol-Myers Squibb, Celltrion, Diadema Farmaceutici, Falk, Fresenius Kabi, Janssen, Merck & Co, Reckitt Benckiser, Regeneron, Sanofi, Shire, SILA, Sofar, Synformulas GmbH, Takeda, Unifarco; he received research support from Reckitt Benckiser, SILA, Sofar, Unifarco; E.M. and V.S. have no disclosures.*

## REFERENCES AND RECOMMENDED READING

Papers of particular interest, published within the annual period of review, have been highlighted as:

▪ of special interest
▪▪ of outstanding interest
